# Consistent effects of pesticides on community structure and ecosystem function in freshwater systems

**DOI:** 10.1038/s41467-020-20192-2

**Published:** 2020-12-10

**Authors:** Samantha L. Rumschlag, Michael B. Mahon, Jason T. Hoverman, Thomas R. Raffel, Hunter J. Carrick, Peter J. Hudson, Jason R. Rohr

**Affiliations:** 1 Department of Biological Sciences, University of Notre Dame, Notre Dame, IN 46556 USA; 2 Department of Integrative Biology, University of South Florida, Tampa, FL 33620 USA; 3 Department of Biology, Miami University, Oxford, OH 45056 USA; 4 Department of Forestry and Natural Resources, Purdue University, West Lafayette, IN 47907 USA; 5 Department of Biological Sciences, Oakland University, Rochester, MI 48309 USA; 6 Department of Biology, Central Michigan University, Mount Pleasant, MI 48859 USA; 7 Huck Institutes of Life Sciences, Pennsylvania State University, State College, PA 16801 USA

**Keywords:** Biodiversity, Ecosystem services, Freshwater ecology, Community ecology

## Abstract

Predicting ecological effects of contaminants remains challenging because of the sheer number of chemicals and their ambiguous role in biodiversity-ecosystem function relationships. We evaluate responses of experimental pond ecosystems to standardized concentrations of 12 pesticides, nested in four pesticide classes and two pesticide types. We show consistent effects of herbicides and insecticides on ecosystem function, and slightly less consistent effects on community composition. Effects of pesticides on ecosystem function are mediated by alterations in the abundance and community composition of functional groups. Through bottom-up effects, herbicides reduce respiration and primary productivity by decreasing the abundance of phytoplankton. The effects of insecticides on respiration and primary productivity of phytoplankton are driven by top-down effects on zooplankton composition and abundance, but not richness. By demonstrating consistent effects of pesticides on communities and ecosystem functions and linking pesticide-induced changes in functional groups of organisms to ecosystem functions, the study suggests that ecological risk assessment of registered chemicals could be simplified to synthetic chemical classes or types and groups of organisms with similar functions and chemical toxicities.

## Abstract (web summary)

The effects of pesticides on individual species could propagate into additional community-wide and ecosystem-level effects. Here the authors use a mesocosm experiment to test how a diverse array of herbicides and insecticides disrupt aquatic community structure and ecosystem function.

## Introduction

Freshwater ecosystems are among the most biodiverse in the world^1^ and provide important ecosystem services^2^, yet many are imperiled by pesticide contamination^3,4^. Two major challenges, among many^5^, impede prediction of responses of freshwater ecosystems to pesticides. First, the extent to which individual pesticides have consistent effects on ecosystem functions and biodiversity is unknown. In the U.S. and Europe, tens of thousands of synthetic chemicals are registered, and in the U.S. >350 pesticides are applied annually^6,7^. If the effects of pesticides are consistent within ‘pesticide classes’ (those with similar chemical structures) or ‘pesticide types’ (those targeting similar pests), then the complexity in predicting impacts of pesticides could be markedly reduced^8,9^. Such consistency would improve efficiency of risk assessment and allow a greater focus on exceptions to general patterns. Second, the role of pesticides in biodiversity-ecosystem function relationships has not been elucidated^10–12^. Historically, random and direct manipulations of single-trophic level communities and measurement of associated ecosystem processes^13,14^ have established causality between biodiversity and ecosystem function^15–17^. However, this approach overlooks the importance of anthropogenic factors (e.g. climate change, nutrient enrichment, pesticide contamination), whose influences on communities are far from random^18,19^, alter multiple trophic levels^14,20^, and occur via direct and indirect pathways^10^.

In an effort to suggest improvements to risk assessment, the objectives of this study were to: (1) evaluate the consistency of effects across pesticide types, classes, and individual pesticides on ecosystem processes and communities, (2) assess whether the effects of pesticides on ecosystem processes and communities were the result of sublethal, non-target effects or changes in abundance of ‘targeted taxa’, and (3) test whether changes in composition, abundance, and/or richness of various functional groups mediate the effects of pesticides on ecosystem functions. We propose three hypotheses. First, ecosystem processes respond consistently to different pesticides within pesticide types because taxonomically related community members often have similar functional roles (redundancy) within the ecosystem. So, reductions in the abundance of taxa of a single group (e.g., green algae) might be specific to an individual pesticide or class, but these reductions would result in similar effects on ecosystem function overall (e.g., primary productivity)^21^. Second, communities respond consistently to pesticides within classes because of taxa-specific sensitivities to pesticides^22,23^. Third, disruptions in ecosystem processes caused by exposure to pesticides are mediated by changes to abundance, composition, and richness of functional groups.

Here, we show that ecosystem functions respond consistently to herbicides and insecticides, while communities respond somewhat less consistently. Pesticide-induced effects on ecosystem functions are driven by changes in the abundance and community composition of functional groups of organisms. For instance, herbicides reduced respiration and primary productivity by decreasing the abundance of phytoplankton, a bottom-up effect. Insecticides increased primary production of phytoplankton and respiration through top-down effects on zooplankton composition and abundance, but not richness. Our results suggest that predictions of the complex effects of pesticides on aquatic ecosystems can be simplified by considering effects of pesticide classes or types on groups of organisms with similar functions and chemical toxicities.

## Results and discussion

### Experimental design

**Fig. 1:**
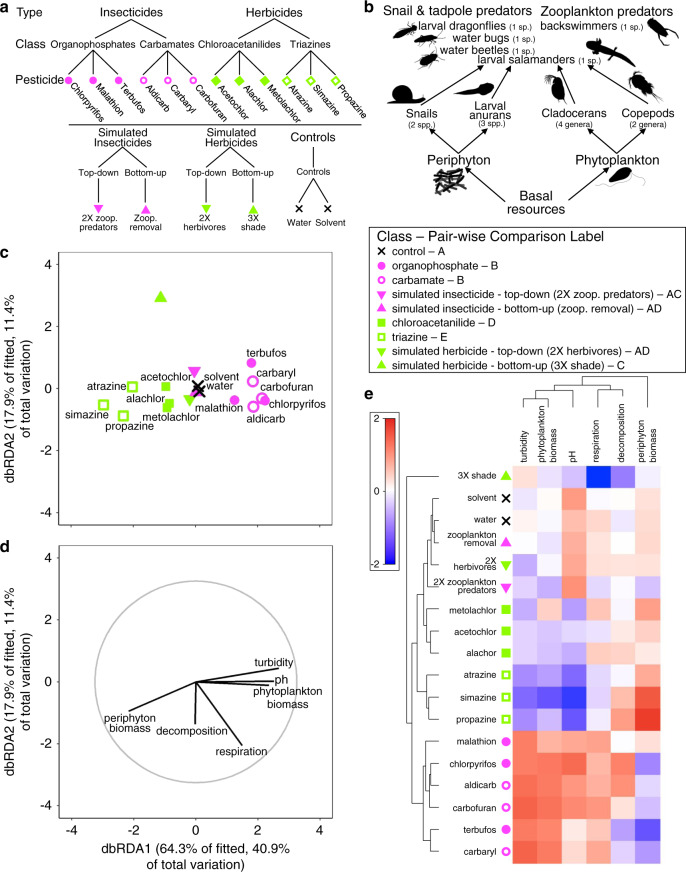
Experimental design and similarity of ecosystem responses by pesticide type. **a** Experimental design showing hierarchical structure of treatments. Each treatment was replicated four times with mesocosm as the replicate. **b** Food web diagram of experimental communities. **c** Distance-based redundancy analysis (dbRDA) plot of multivariate ecosystem responses showing differences among treatments grouped by pesticide type. Individual points are the centroids of 18 treatments in the experiment. For pair-wise comparisons, treatments sharing letters are not different from each other. **d** Vector overlay of ecosystem responses for the corresponding dbRDA plot. The gray circle corresponds to vector lengths that would have a correlation coefficient of one with each axis. **e** Cluster diagram of experimental treatments and ecosystem-level responses showing grouping of experimental treatments according to pesticide type. The scale bar shows the magnitude of the effect of a treatment on an individual response. The values of the colors correspond to the averaged and normalized treatment responses (see “Methods” section). Attribution of silhouettes: periphyton (created by Matt Crook, license link, image has been rotated), anuran tadpole (created by M. Mahon (vectorization), J.J. Harrison (photography), photo license link, photo changed to silhouette), *A. junius* (created by M. Mahon (vectorization), Dave Huth (photography), photo license link, photo changed to silhouette), *B. flumineum* (created by Dave Angelini, license link, image was rotated), and *Hydrochara* (created by T. Michael Keesey (vectorization), Yves Bousquet (photography), license link, image was rotated).

While we recognize that an enormous challenge to predicting the effects of synthetic chemicals on complex natural systems is to understand the effects of mixtures of synthetic chemicals, this study focuses on the effects of single pesticides because scientists do not yet fully understand the effects of even single pesticides on complex ecosystems^3,24–26^. In addition, federal agencies are charged with evaluating ecological safety of synthetic chemicals one at a time as they come to market or need to be re-evaluated. Therefore, a framework must first be developed for understanding the effects of single synthetic chemicals before reliable predictions can be developed for the responses of communities and ecosystems to chemical mixtures^27^. Mesocosm studies are an efficient approach to toxicity testing as they provide toxicity data on multiple species simultaneously under environmentally realistic conditions. As such, we conducted a large-scale experiment using 72 outdoor mesocosms to evaluate the effects of two control treatments (water and solvent), four simulated-pesticide treatments, and 12 pesticides on tri-trophic temperate pond communities (Fig. 1a, b). The pesticide treatments were nested in four classes (organophosphates, carbamates, chloroacetanilides, and triazines) and two types (insecticides and herbicides) (Fig. 1a). The four pesticide classes in this study are representative of some of the most commonly used^7^ and detected pesticide classes in the US^28,29^. To represent pesticide runoff following rainfall, pesticides were applied singly at the beginning of the experiment at standardized environmentally relevant concentrations calculated using U.S. EPA software’s GENEEC v2 (see “Methods” section). Simulated-pesticide treatments were top-down or bottom-up food web manipulations that attempted to mimic direct (i.e. lethal) effects of actual herbicides and insecticides on algae and zooplankton abundances, respectively.

### Effects of pesticide types on ecosystem function

Pesticide type explained 46% of the variation in ecosystem function associated with the pesticide treatments (Supplementary Table 1). Herbicides were associated with a decrease in primary production of phytoplankton that led to increased production of attached periphyton (benthic algal biofilms), probably through an increase in light availability (Fig. 1c–e). In addition, as production of phytoplankton decreased in response to herbicide exposure, respiration decreased. (Fig. 1c-e). These patterns are described in more detail in a structural equation model below. Herbicide exposure also lead to a decrease in pH (an increase in acidity), which might reflect the release of dissolved inorganic carbon as a result of decomposing phytoplankton.

In contrast to herbicide-exposed systems, insecticide-exposed systems exhibited an increase in production of phytoplankton, whose growth in the water column reduced light penetration, thereby shading and reducing the primary production and biomass of benthic periphyton (Fig. 1c–e), an effect shown in other studies^22^. Increases in phytoplankton were likely driven by replacement of cladocerans by copepod zooplankton, the latter of which are less efficient phytoplankton grazers (described below). The corresponding increase in phytoplankton also lead to a subsequent increase in respiration (Fig. 1c–e). As phytoplankton production increased, pH increased (acidity decreased), a possible result from an increase in phytoplankton removing inorganic carbon from the water column. While some variation in ecosystem responses was also explained by individual pesticides, it was small relative to variation explained by pesticide type (12 vs. 46% of variation in ecosystem responses, Supplementary Table 1). Decomposition of leaf litter did not appear to be strongly influenced by either herbicides or insecticides (Fig. 1c–e).

### Effects of pesticide types and classes on communities

**Fig. 2:**
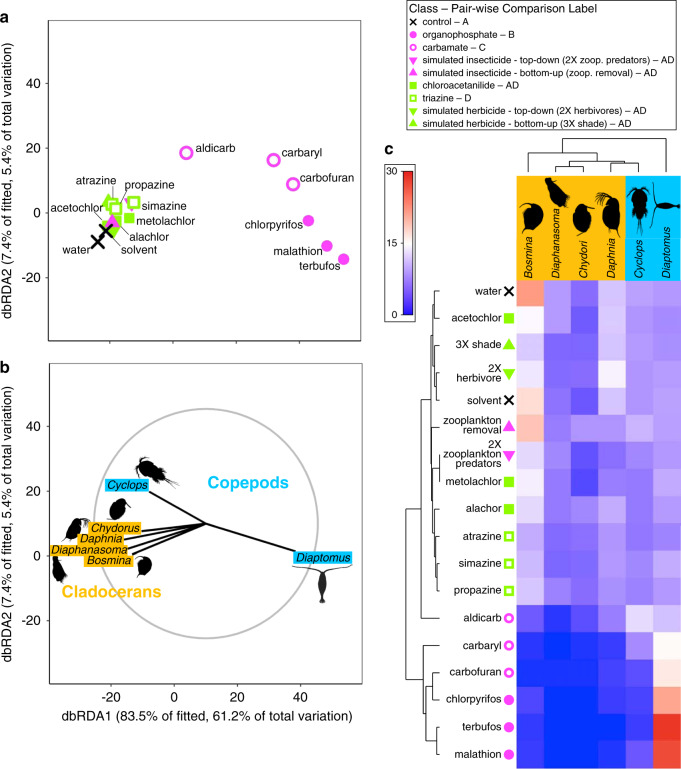
Zooplankton communities respond consistently to pesticides within type, class, and individual pesticide. **a** Distance-based redundancy analysis (dbRDA) plot of multivariate zooplankton densities by genera showing differences among treatments grouped by type, class, and individual pesticide. Individual points correspond to the 18 treatments in the experiment. For pair-wise comparisons, treatments sharing letters are not different from each other. **b** Vector overlay of zooplankton responses for the corresponding dbRDA plot. The gray circle corresponds to vector lengths that would have a correlation coefficient of one with each axis. **c** Cluster diagram of experimental treatments and zooplankton densities showing differences among type, class, and individual pesticide. The scale bar shows the magnitude of the effect of a treatment on an individual response. The values of the colors correspond to the averaged and normalized treatment responses (see “Methods” section).

We tested for the effects of individual pesticides, classes, and types separately on the single-trophic-level zooplankton community (six zooplankton genera) and on the tri-trophic community (insect and salamander predators, snail and anuran herbivores, and periphyton and phytoplankton primary producers). Similar to ecosystem function, pesticide type explained the majority of the variance (44.2%) in the zooplankton community, followed by pesticide class (18.8%) (Supplementary Table 1). Distance-based redundancy analysis (dbRDA) showed that: (1) herbicide-treated and insecticide-treated mesocosms had distinct zooplankton communities, (2) within their respective pesticide types, organophosphate insecticides, chloroacetanilide herbicides, and triazine herbicides caused further distinction in zooplankton communities, and (3) there was relatively high multivariate dispersion within the carbamate class (Fig. 2a, b). In response to insecticides, cladoceran zooplankton experienced high mortality and were virtually eliminated, which perhaps led to competitive release of copepods (Fig. 2a–c)^30^. Cladocerans are more efficient phytoplankton grazers than copepods^31^, so it stands to reason that their declines potentially drove an increase in the relative abundance of phytoplankton in these treatments (Fig. 1). In contrast to the changes in community composition associated with insecticides, herbicides decreased zooplankton abundance with no apparent change in composition (Supplementary Fig. 3), likely by reducing phytoplankton (i.e., bottom-up effect^32^,). The stronger bottom-up effect of triazines compared to chloroacetanilide herbicides on zooplankton was probably because of longer environmental persistence (soil half-lives 110–146 days vs. 14–26 days, respectively [Pesticide Action Network Pesticide Database]). Thus, consistent with the ecosystem function results, these findings on the zooplankton community suggest that ecological risk assessment can be largely simplified to generalized effects of pesticide type or class.

**Fig. 3:**
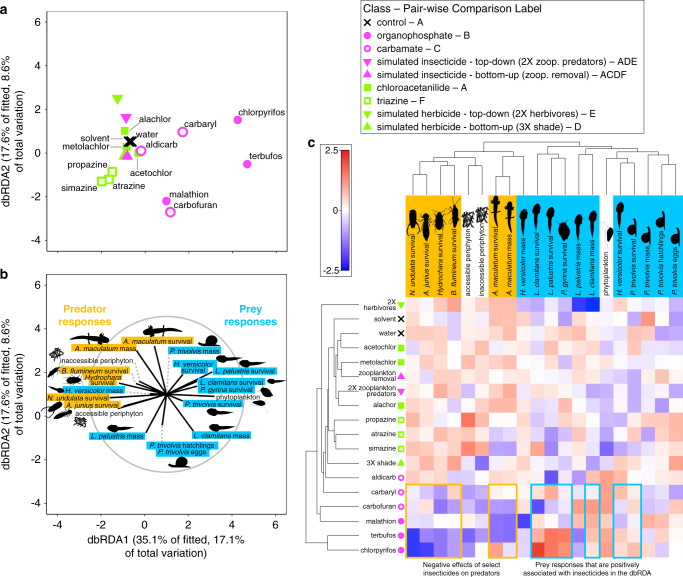
Insecticides generally reduce insect predators in tri-trophic communities, increasing the survival and growth of their prey. **a** Distance-based redundancy analysis (dbRDA) plot of multivariate community responses showing differences among treatments grouped by type, class, and individual pesticide. Individual points correspond to the 18 treatments in the experiment. For pair-wise comparisons, treatments sharing letters are not different from each other. **b** Vector overlay of community responses for the corresponding dbRDA plot. The gray circle corresponds to vector lengths that would have a correlation coefficient of one with each axis. **c** Cluster diagram of experimental treatments and community responses, showing differences among type, class, and individual pesticide. The scale bar shows the magnitude of the effect of a treatment on an individual response. The values of the colors correspond to the averaged and normalized treatment responses (see “Methods” section). When responses within tri-trophic communities are grouped by functional role (algae, herbivores, predators), type explains twice as much variation as class and individual pesticide (Supplementary Table 1). Attribution of silhouettes: periphyton (created by Matt Crook, license link, image has been rotated), anuran tadpole (created by M. Mahon (vectorization), J.J. Harrison (photography), photo license link, photo changed to silhouette), *A. junius* (created by M. Mahon (vectorization), Dave Huth (photography), photo license link, photo changed to silhouette), *B. flumineum* (created by Dave Angelini, license link, image was rotated), *Hydrochara* (created by T. Michael Keesey (vectorization), Yves Bousquet (photography), license link, image was rotated), and *P. gyrina* (created by M. Mahon (vectorization), N. Yotarou (photography), photo license link, photo changed to silhouette).

In the tri-trophic community, variation explained by pesticides was about equally distributed among type, class, and individual pesticide (Supplementary Table 1). The dbRDA showed that: (1) herbicide-treated and insecticide-treated mesocosms had distinct responses of community members, (2) within herbicides, triazines classes caused further distinction in communities, and (3) there was relatively high multivariate dispersion in communities exposed to carbamate and organophosphate insecticides (Fig. 3a, b). Overall, survival of predators was low with insecticides^33^, except for aldicarb (Fig. 3a-c). Amphibian and snail prey generally had greater positive responses to insecticides compared to controls or herbicides (Fig. 3a-c), suggesting these organisms benefitted from predatory release, a trend found in other studies^34^. While multigenerational exposure of organisms to pesticides has the potential to alter vulnerability of organisms over time, it seems unlikely that it played a large role in our study given the length of the experiment, the persistence of the pesticides, and the generation time of the focal organisms of study (see Supplementary Discussion). In addition, none of the patterns on community or ecosystem-level responses could be explained by structural similarities among pesticide classes (see Supplementary Discussion).

Next, we used physical manipulation of communities in the application of simulated pesticide treatments in order to evaluate how direct vs. indirect effects of pesticides on aquatic communities influenced ecosystem functions. However, rarely were these treatments similar to actual pesticide classes (pair-wise comparisons Figs. 1–3). This lack of similarity was presumably because of difficulties in sustaining manipulations in the simulated pesticide treatments that matched the magnitude and specificity of actual pesticides on species with short generation times (see Supplementary Discussion and Supplementary Fig. 3 for details). While manipulating species composition has been critical historically in the study of biodiversity-ecosystem function, the same approaches are not well-suited for (1) species that can exhibit population dynamics in the timescale of an experiment because they may quickly recover from a manipulation and (2) studies of disturbance because matching the complexity of the effects of actual disturbance is challenging.

### Linking pesticide induced changes in communities to ecosystem function

**Fig. 4:**
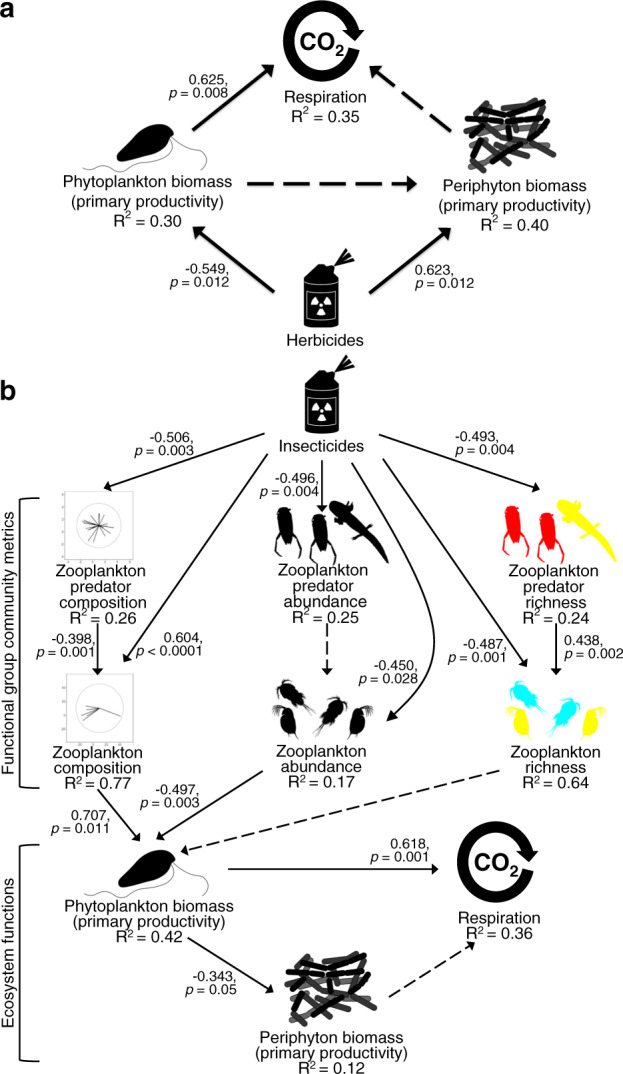
Structural equation models linking pesticides-induced changes in composition, abundance, and richness of functional groups to ecosystem functions. **a** Through bottom-up effects, herbicide exposure decreases respiration through decreases in the abundance of phytoplankton. **b** The effects of insecticides on respiration and primary production of phytoplankton are driven by top-down effects on zooplankton composition and abundance, but not richness. Solid arrows are significant paths, and dotted arrows are non-significant paths. *P*-values, standardized coefficients, and conditional *R*^2^ values are provided. In **b**, the residuals of zooplankton composition, abundance, and richness covary, and the residuals of zooplankton predator composition, abundance, and richness covary. In both structural equation models, individual paths were linear models. Individual path *P*-values were based two-sided t-tests. The data fit the models well (**a**: Fisher’s C = 3.601 with *P*-value = 0.165 on 2 degrees of freedom, **b**: Fisher’s C = 53.176 with *P*-value = 0.282 on 48 degrees of freedom). Attribution of silhouette: periphyton (created by Matt Crook, license link, image has been rotated).

Finally, we used structural equation models acting on the natural variation created by pesticide exposures to evaluate how composition, abundance, and richness of different functional groups of organisms influenced whole-system respiration and primary production of phytoplankton and periphyton. Our results show that herbicide exposure decreased respiration and primary productivity through bottom-up effects; herbicides decreased the abundance of phytoplankton, which in turn drove respiration (Fig. 4a). In contrast, insecticides increase primary productivity of phytoplankton and respiration through top-down effects on zooplankton composition and abundance, but not richness (Fig. 4b).

Given that ecosystem functions respond more consistently to pesticides than communities and pesticide-induced changes to ecosystem functions were at times mediated by changes in abundance and composition of functional groups of organisms, the observed consistency in the responses of ecosystem functions to pesticides within pesticide types could be driven by functional redundancies of species. Further supporting this point, when we grouped responses of taxa by their functional roles in the community (algae, herbivores, and predators), the variance explained by pesticide type (29%) was nearly doubled compared to the variance accounted for by either pesticide class (17.6%) or individual pesticide (17.3%) (Supplementary Table 1). These results suggest that the complexity in predicting the effects of pesticides on communities could be reduced by evaluating responses of functional groups to pesticide classes or types rather than evaluating the responses of species or genera to individual pesticides. While this outcome presents an opportunity to simplify predictions, it also is a sobering illustration of the blanket detrimental effects pesticides can have on complex ecological systems.

Our results suggest that ecological risk assessment by regulatory agencies, made complex by tens of thousands of synthetic chemicals and diverse species assemblages, could be advanced in two ways. First, regulators could extend this study to additional pesticides and develop predictions based on consistencies of effects within chemical classes or types^25^. Additional pesticides of different types and classes might produce different sequences of direct and indirect effects of varying magnitudes on aquatic ecosystems. Nonetheless, we predict that, on average, ecosystem and community effects would still be consistent within pesticide classes and types, with more variation being explained by types. The generality of effects by class and type would be driven by the nestedness of the pesticides’ biologic activity; molecular modes of action are shared within classes (e.g., carbamates inhibit acetylcholinesterase) and targets in the environment are shared within types (e.g., insecticides are active against insects). A similar approach of using chemical structures of synthetic chemicals to predict toxicities is taken by Quantitative Structure-Activity Relationships (QSAR) analyses^35^. QSAR analyses on the present herbicides and insecticides did not recreate the same groupings of pesticides by class and by type as the current analyses (see Supplementary Discussion). The QSAR results were likely not similar to the present results because QSAR analyses are based on predictions from model organisms, which assumes that the responses of the model species are correlated with responses of all organisms that they are selected to represent. This assumption is problematic because of taxa-specific sensitivities to synthetic chemicals. In addition, QSAR analyses ignore species interactions and indirect effects, which we show are important considerations for predicting community-level and ecosystem-level responses.

Second, regulators could extend this study to additional communities and ecosystems and develop predictions based on the consistencies of effects within taxonomic or functional groups of organisms. Testing synthetic chemicals against entire communities and ecosystems incorporates natural complexity and allows for the evaluation of both direct and indirect effects. While we observed that organisms within functional groups organisms share similar direct and indirect toxicities to pesticide classes and types, it is possible that the genetic makeup^36^, community complexity and composition^37^, presence of other stressors^38^, and previous exposure history^39^ could at times obscure the generality of the effects of pesticides when additional communities and ecosystems are considered. Nevertheless, the addition of standardized tests across communities and ecosystems would allow regulators to assess just how important these factors are to their regulatory decisions. Although we fully expect that there could be occasional exceptions to the general patterns revealed and proposed in this study, by simplifying risk assessment in the ways we suggest, more time and resources would be available from regulatory agencies to detect any exceptions to these general patterns.

## Methods

### Experimental design and community composition

We conducted a randomized-block experiment at the Russell E. Larsen Agricultural Research Center (Pennsylvania Furnace, PA, USA) with replicated mesocosm ponds. Mesocosms were 1100-L cattle tanks covered with 60% shade cloth. The spatial block was distance from a tree line in our mesocosm field. Three weeks before pesticide application, these mesocosms were filled with 800 L water, 300 g mixed hardwood leaves, and inoculations of zooplankton, periphyton, and phytoplankton homogenized from four local ponds. Just before pesticide application on the same day, each tank received two snail, three larval anuran, one larval dragonfly, one water bug, one water beetle, one larval salamander, and one backswimmer species (11 *Helisoma* (*Planorbella*) *trivolvis*, 10 *Physa gyrina*; 20 *Hyla versicolor*, 20 *Lithobates palustris*, 20 *Lithobates clamitans*; 2 *Anax junius*; 2 *Belostoma flumineum*; 5 *Hydrochara* sp.; 3 *Ambystoma maculatum*; 6 *Nototeca undulata*) (Fig. 1b). These community members naturally coexist and were applied at naturally occurring densities^40^. Initial conditions of some mesocosms varied in simulated pesticide treatments (see below).

We randomly assigned 18 treatments (12 pesticides, 4 simulated pesticides, 2 controls) with four replicate mesocosms of each treatment, which resulted in 72 total mesocosms (Fig. 1a). The 12 pesticide treatments were nested; we included two pesticide types (insecticide, herbicide), two classes within each pesticide type (organophosphate insecticide, carbamate insecticide, chloroacetanilide herbicide, triazine herbicide), and three different pesticides in each of four classes (Fig. 1a). To represent runoff of pesticides into freshwater systems following a rainfall event, we applied single doses of technical grade pesticides at environmentally relevant concentrations at the beginning of the experiment. To ensure our exposures represented environmental relevance, we used estimated environmental concentrations of pesticides, calculated by U.S. Environmental Protection Agency’s GENEEC v2 software, Supplementary Table 2). Our design also included water and solvent (0.0001% acetone) controls (Fig. 1a). Pesticides were obtained from ChemService (West Chester, PA, USA). Nominal concentrations of pesticides (μg/L) were: 64 chlorpyrifos, 101 malathion, 171 terbufos, 91 aldicarb, 219 carbaryl, 209 carbofuran, 123 acetochlor, 127 alachlor, 105 metolachlor, 102 atrazine, 202 simazine, and 106 propazine. We collected composite water samples 1 h after application to mesocosms and shipped samples on ice to Mississippi State Chemical Laboratory to verify these nominal concentrations. Measured concentrations of pesticides (μg/L) were: 60 chlorpyrifos, 105 malathion, 174 terbufos, 84 aldicarb, 203 carbaryl, 227 carbofuran, 139 acetochlor, 113 alachlor, 114 metolachlor, 117 atrazine, 180 simazine, and 129 propazine.

The four simulated pesticide treatments were top-down or bottom-up food web manipulations intended to mimic effects of actual herbicides and insecticides on community members. These manipulations occurred once and were concurrent with the timing of pesticide applications. Top-down and bottom-up simulated insecticide treatments were designed to reduce densities of zooplankton, simulating effects of insecticides on zooplankton survival. For top-down simulated insecticides, we doubled the densities of zooplankton predators by including six total *A. maculatum* larval salamanders and 12 *N. undulata* backswimmers per mesocosm. For bottom-up simulated insecticides (i.e., direct manipulation of a lower arthropod trophic level), we removed zooplankton with a net. Top-down and bottom-up simulated herbicides were designed to reduce algae, simulating effects of herbicides on survival and growth of algae. For top-down simulated herbicides, we doubled the densities of large herbivores to increase grazing pressure by including 22 *H. trivolvis* snails, 20 *P. gyrina* snails, 40 *H. versicolor* larval anurans, 40 *L. palustris* larval anurans, and 40 *L. clamitans* larval anurans per mesocosm. For bottom-up simulated herbicides, we covered mesocosms in three sheets of 60% shade cloth in an attempt to block light and reduce photosynthesis. The experiment ran for four weeks, from June to July.

### Measurements of experimental responses

During the experiment, we sampled periphyton using clay tiles (100 cm^2^) oriented perpendicularly along the bottom of the mesocosm. Each mesocosm had two periphyton measurements: ‘inaccessible periphyton’ taken from caged clay tiles that excluded herbivores and ‘accessible periphyton’ taken from clay tiles that were uncaged, allowing herbivore access. We sampled phytoplankton from water samples taken 10 cm below the water surface. Periphyton was scrubbed from tiles and phytoplankton from water samples (10 mL) were filtered onto glass fiber filters (under low vacuum pressure, <10 psi; Whatman EPM 2000, 0.3 μm, 47 mm) to estimate associated chlorophyll concentrations. The chlorophyll concentration of each filter was determined using an organic extraction procedure with a 50:50 mixture of 90% acetone to DMSO. We measured chlorophyll-*a* concentrations using a standard fluorometric technique. We scored water clarity, a metric of light availability, on a scale from one (clear) to five (opaque) blinded to treatment. We measured pH and dissolved oxygen (DO) at dusk and dawn on subsequent days using hand-held meters (YSI, Yellow Springs, OH, USA). We measured decomposition by taking the dry mass of hardwood leaf packets in each mesocosm at the beginning and the end of experiment. In addition, we sampled snail egg masses and hatchlings using two rectangular pieces of Plexiglass (465 cm^2^) in each mesocosm, one hung on the side and one on the bottom of the mesocosm. Zooplankton were collected from the entire water column by placing a PVC pipe (10 cm diameter, 60 cm height) upright in the center of each tank, capping the bottom, and pouring the water through a 20 μm Nitex mesh. We collected two samples of zooplankton from each mesocosm, and we combined and preserved the samples in 70% ethanol. Zooplankton were counted and identified in 5 mL subsamples for each mesocosm using a zooplankton counting wheel (Wildlife Supply Company, Yulee, FL, USA) and a dissecting microscope. At the end of the experiment, mesocosms were drained, and the remaining animals were counted, euthanized, and preserved. Two previous manuscripts, which use the same design as the current manuscript, also describe this experimental design and methods^8,41^. The research was reviewed and approved by the Penn State University Institutional Animal Care and Use Committee.

### Statistical analyses

To test for the consistency of effects of type, class, and individual pesticide on aquatic ecosystem processes and communities and to attribute the variation explained to each pesticide level of organization while accounting for the nested structure of our experimental design (Fig. 1a), we completed permutational analyses of variance (PERMANOVA). For nested PERMANOVA models, the predictors were the following random categorical terms: type (insecticide, herbicide), class (carbamate, organophosphate, chloroacetanilide, triazine) nested within type, and pesticide (12 in total) nested within class within type. These models did not include controls or simulated pesticides because these treatments were not hierarchically nested (Fig. 1a). We evaluated 9999 permutations using residuals under a reduced model. Following nested PERMANOVAs, we used pair-wise multiple comparisons tests using PERMANOVAs to evaluate differences among controls, organophosphates, carbamates, top-down simulated insecticides, bottom-up simulated insecticides, chloroacetanilides, triazines, top-down simulated herbicides, and bottom-up simulated herbicides. In these pair-wise comparisons, we evaluated 9999 unrestricted permutations of raw data. All PERMANOVAs also included spatial block as a random predictor to account for variation in sunlight associated with distance from a tree line. Preliminary analyses showed that exclusion of the block did not change the results. In all PERMANOVAs, test statistics associated with Type III partial sums of squares were evaluated.

We conducted four nested PERMANOVAs. Our first nested PERMANOVA focused on ecosystem processes and included the following responses: pH taken at dawn, respiration (the difference between dissolved oxygen at dusk and dissolved oxygen at dawn of the subsequent day), decomposition (percent mass remaining of hardwood leaf packets), turbidity (water clarity scores from 1 to 5), and densities of phytoplankton and accessible periphyton (measured via chlorophyll-*a*). Since this analysis was focused on ecosystem-level responses, for periphyton we include accessible periphyton and not inaccessible periphyton because accessible periphyton better encompasses the totality of biologic (e.g. herbivore predation, shading from phytoplankton) factors that could influence periphyton biomass at the ecosystem level. Preliminary analyses included both accessible and inaccessible periphyton, and the results did not differ. The resemblance matrix for these responses was constructed using a Euclidean distance matrix of log-transformed and normalized values.

Our second and third nested PERMANOVAs focused on community structure. We separated community members into two statistical models based on the forms of response variables; those whose response variables were densities based on counts (zooplankton) and those community members whose response variables were survival, mass, reproductive rates, or density abstracted from chlorophyll measurements (insect predators, snail and tadpole herbivores, and algae; termed the tri-trophic community). The multivariate response for the zooplankton community included densities of *Daphnia*, *Diaphanasoma*, *Chydorus*, *Bosmina*, *Diaptomus*, and *Cyclops*. Zooplankton community analyses were based on square-root transformed densities using Bray-Curtis similarities. The multivariate response for the tri-trophic community model included: survival (0 to 1) of all amphibian, snail, and insect community members; average masses of surviving individuals for each amphibian species and *H. trivolvis* snails; average number of hatchlings and eggs per surviving *H. trivolvis* snail; and densities of phytoplankton and accessible and inaccessible periphyton. In this analysis, we include both accessible and inaccessible periphyton to account for any differential effects of excluding herbivores on the abundance of periphyton. Mass and reproductive rates were standardized to the number of surviving individuals to account for the different densities added to each tank at the beginning of the study (i.e. extra herbivores in top-down simulated herbicide treatment and extra predators in bottom-up simulated insecticide treatment). Finally, our fourth nested PERMANOVA evaluated a simplified tri-trophic community. We simplified the tri-trophic community responses into three functional roles within the community: algae, herbivores, and predators. Tri-trophic community responses of individua taxa were transformed and normalized as described previously, and then they were averaged according to functional group. We averaged densities of periphyton and phytoplankton into a single “algae” response, all amphibian and snail responses into a single “herbivore” response, and all insect and salamander responses into a single “predator” response. The simplified tri-trophic community model was based on Euclidean distances.

To visualize consistency of effects within type, class, and individual pesticides on multivariate ecosystem and community responses and to compare pesticide effects to simulated pesticides and controls, we used distance-based redundancy analyses (dbRDA) and two-way cluster diagrams. The dbRDAs were based on appropriate resemblance matrices for ecosystem and community responses as described above. The underlying categorical predictors in all models included: the spatial block, organophosphate, carbamate, chloroacetanilide, triazine, top-down simulated insecticide, bottom-up simulated insecticide, top-down simulated herbicide, bottom-up simulated herbicide, and control. In the dbRDA plots, we show the centroid values for the 18 experimental replicates.

As an alternative to the dbRDAs presented in the main text, we also visualized the consistency of effects within type, class, and individual pesticide on ecosystem, tri-trophic community, and zooplankton responses and compared pesticide effects to simulated pesticides and controls, using principal coordinates analyses (PCoA) (Supplementary Figs. 1, 2, 4). PCoAs were based on appropriate resemblance matrices as described previously. PCoAs were conducted in PERMANOVA+ for PRIMER and resulting data were exported. Point and vector plots were made using the exported data and the ‘*ggplot2*’ package in R. Ellipses on point plots represent 95% confidence intervals of groups based on standard errors and were made using the ordiellipse function in the ‘*vegan*’ package.

For the two-way cluster diagrams, clusters of pesticide treatments were based on centroid distances of the appropriate resemblance matrices. Clusters of multivariate responses were based on Euclidean distance resemblance matrices of averaged treatment responses. Before averaging, ecosystem and community responses were transformed and normalized as described previously. In clustering of treatments and responses, the cluster mode was the group average. In the PERMANOVAs for tri-trophic community responses, the effect of block was significant (Supplementary Table 1). Thus, we accounted for the effect of block by taking the residuals of simple linear regressions with individual tri-trophic community responses as the independent variable and block as the predictor in the generation of the shaded values of the two-way cluster diagrams. Then, we averaged these block-adjusted treatment responses with the ‘shade plot’ function in PRIMER. For the ecosystem responses and zooplankton community, the effect of block was not significant in the PERMANOVA model (Supplementary Table 1), so shaded values of the two-way cluster diagrams were simply the averaged treatment responses. All PERMANOVA models, pair-wise comparisons, dbRDAs, and two-way cluster diagrams were executed using PERMANOVA+ for PRIMER version 7 (PRIMER-E Ltd, Plymouth, UK). For ease of visualization of dbRDA and PCoA plots, data from PERMANOVA+ for PRIMER were exported, and plots were made using ‘*ggplot2*’ package in R.

To quantitatively test whether changes in composition, abundance, and/or richness of different functional groups mediate the effects of herbicides and insecticides on ecosystem function, we performed structural equation modeling and model comparison using the ‘*piecewiseSEM’* package. We conducted separate analyses for insecticides and herbicides. Global models, which replicated the structures of periphyton and phytoplankton food webs (Fig. 1b), evaluated how herbicides or insecticides influence ecosystem functions through changes in composition, abundance, and richness of: (1) snail and tadpoles predators, (2) snails and tadpoles, (3) zooplankton predators, and (4) zooplankton. Ecosystem functions included primary production of accessible periphyton, primary production of phytoplankton, and whole system respiration. We did not include decomposition because ordination analyses showed decomposition was not dramatically influenced by pesticide exposures. Since this analysis was focused on ecosystem-level responses, we included accessible periphyton only (i.e. excluded inaccessible periphyton) because accessible periphyton better encompasses the totality of biologic (e.g. herbivore predation, shading from phytoplankton) factors that could influence periphyton biomass at the ecosystem level. For each functional group, composition was the locations of species or genera along the first axis of a principal coordinates analysis (PCoA) using a Bray-Curtis similarity matrix. Abundance was the total number of individuals of a functional group in a mesocosm, and richness was the number of species or genera within a functional group. The relationships among these variables in the global models are presented in Supplementary Fig. 5. Notably for herbicides, the global model considered the bottom-up direct effects of herbicides on periphyton and phytoplankton and the non-target direct effects of herbicides on zooplankton composition, abundance, and richness. Mesocosms treated with chloroacetanilide herbicides were excluded from the analysis because they did not influence algal densities (Fig. 3c). For insecticides, the global model considered the top-down direct effects of insecticides on composition, abundance, and richness of tadpole and snail predators, zooplankton predators, and zooplankton. These global models did not consider non-target direct effects of insecticides and herbicides on tadpoles and snails because community tri-trophic community analyses (Fig. 3) showed little evidence of non-target effects on these organisms. Phytoplankton, periphyton, and abundance of functional groups were all log-transformed in models. To facilitate comparisons among responses and clarify relationships among predictors, we reduced the global models by removing non-significant paths. The final models are presented in Fig. 4. All data supporting the results of the present manuscript are publicly available^42^. Full information regarding creation and attribution of silhouettes used throughout the main text and supplementary figures is included in Supplementary Table 3.

### Reporting summary

Further information on research design is available in the Nature Research Reporting Summary linked to this article.

## Supplementary information

Supplementary Information

Reporting Summary

## Data Availability

Data used in the generation of pesticide concentrations used in the present experiment are available within supplementary information. All other relevant data supporting the findings of this study are available at Figshare (10.6084/m9.figshare.13224587).
