# Publisher Correction: Consistent effects of pesticides on community structure and ecosystem function in freshwater systems

**DOI:** 10.1038/s41467-020-20854-1

**Published:** 2021-01-14

**Authors:** Samantha L. Rumschlag, Michael B. Mahon, Jason T. Hoverman, Thomas R. Raffel, Hunter J. Carrick, Peter J. Hudson, Jason R. Rohr

**Affiliations:** 1 Department of Biological Sciences, University of Notre Dame, Notre Dame, IN 46556 USA; 2 Department of Integrative Biology, University of South Florida, Tampa, FL 33620 USA; 3 Department of Biology, Miami University, Oxford, OH 45056 USA; 4 Department of Forestry and Natural Resources, Purdue University, West Lafayette, IN 47907 USA; 5 Department of Biological Sciences, Oakland University, Rochester, MI 48309 USA; 6 Department of Biology, Central Michigan University, Mount Pleasant, MI 48859 USA; 7 Huck Institutes of Life Sciences, Pennsylvania State University, State College, PA 16801 USA

**Keywords:** Biodiversity, Ecosystem services, Freshwater ecology, Community ecology

Correction to: *Nature Communications* 10.1038/s41467-020-20192-2, published online 10 December 2020.

The original version of this Article contained an error in Fig. 4, in which the incorrect colours were reproduced. The correct version of Fig. 4 is:



which replaces the previous incorrect version:



This has now been corrected in both the PDF and HTML versions of the Article.

