# Supplementary material for: Consistent effects of pesticides on community structure and ecosystem function in freshwater systems
Source: Nat Commun. 2020 Dec 10;11:6333. doi: 10.1038/s41467-020-20192-2 (PMC7730384; doi:10.1038/s41467-020-20192-2)
Supplement: Supplementary file 1 — Supplementary Information [file 41467_2020_20192_MOESM1_ESM.pdf]

Consistent effects of pesticides on community structure and ecosystem function in freshwater systems

Samantha L. Rumschlag*, Michael B. Mahon, Jason T. Hoverman, Thomas R. Raffel, Hunter J. Carrick, Peter J. Hudson, Jason R. Rohr

*correspondence to: srumschl@nd.edu

This PDF file includes:

Supplementary Discussion
Supplementary Figures 1 to 6
Supplementary Tables 1 to 3

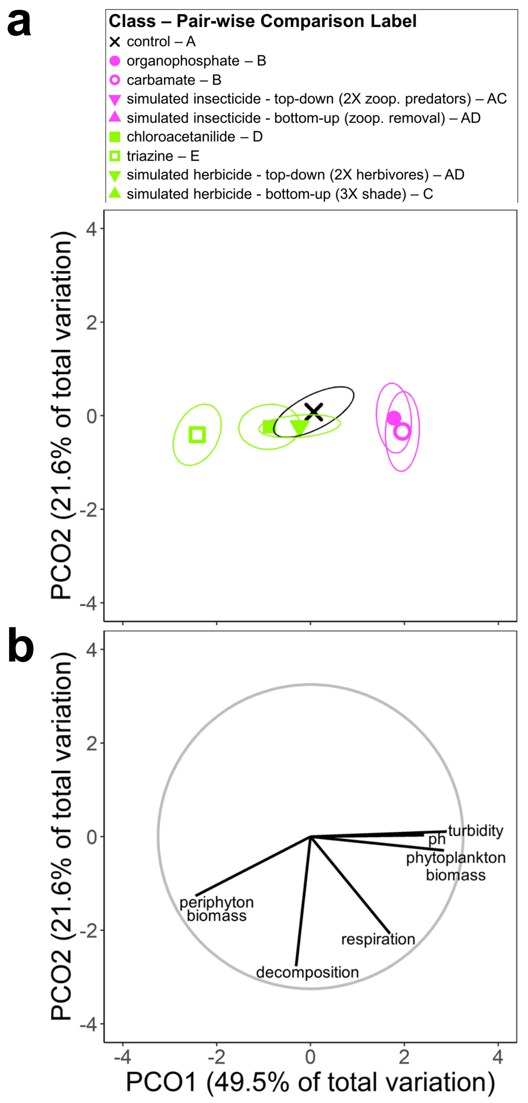

Supplementary Figure 1. Principal coordinates analysis for multivariate ecosystem responses. a Principal coordinates analysis plot of multivariate ecosystem-level responses, showing differences in pesticide treatments by type. Individual points are centroids for the representative treatment with ellipses based on 95% confidence intervals calculated using a standard error. Of all the simulated pesticide treatments, only top-down simulated herbicide was not different from an actual herbicide class. So, all other simulated pesticide treatments were not included in the plot for ease of viewing. Pair-wise comparison labels are given in the figure legend. Treatments sharing letters are not different from each other. **b** Vector overlay of log-transformed and normalized ecosystem-level responses for corresponding principal coordinates analysis plot. Gray circle shows relative vector distance lengths; the gray circle corresponds to vector lengths that would have a correlation coefficient of one.

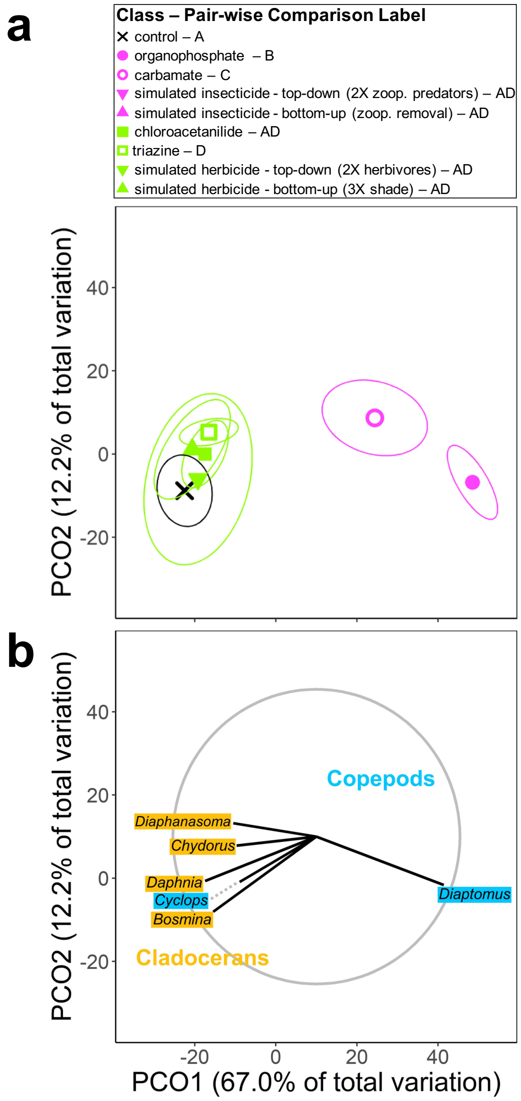

Supplementary Figure 2. Principal coordinates analysis for multivariate zooplankton responses. a Principal coordinates analysis plot of multivariate zooplankton densities by genera, showing differences in pesticide treatments by type, class, and individual pesticide. Individual points are centroids for the representative treatment with ellipses based on 95% confidence intervals calculated using a standard error. Simulated pesticides that were different from corresponding pesticide treatments, including bottom-up and top-down simulated insecticides, were not included in the plot for ease of viewing. Pair-wise comparison labels are given in the figure legend. Treatments sharing letters are not different from each other. **b** Vector overlay of square-root transformed zooplankton densities by genera for corresponding principal coordinates analysis plot. Gray circle shows relative vector distance lengths; the gray circle corresponds to vector lengths that would have a correlation coefficient of one.

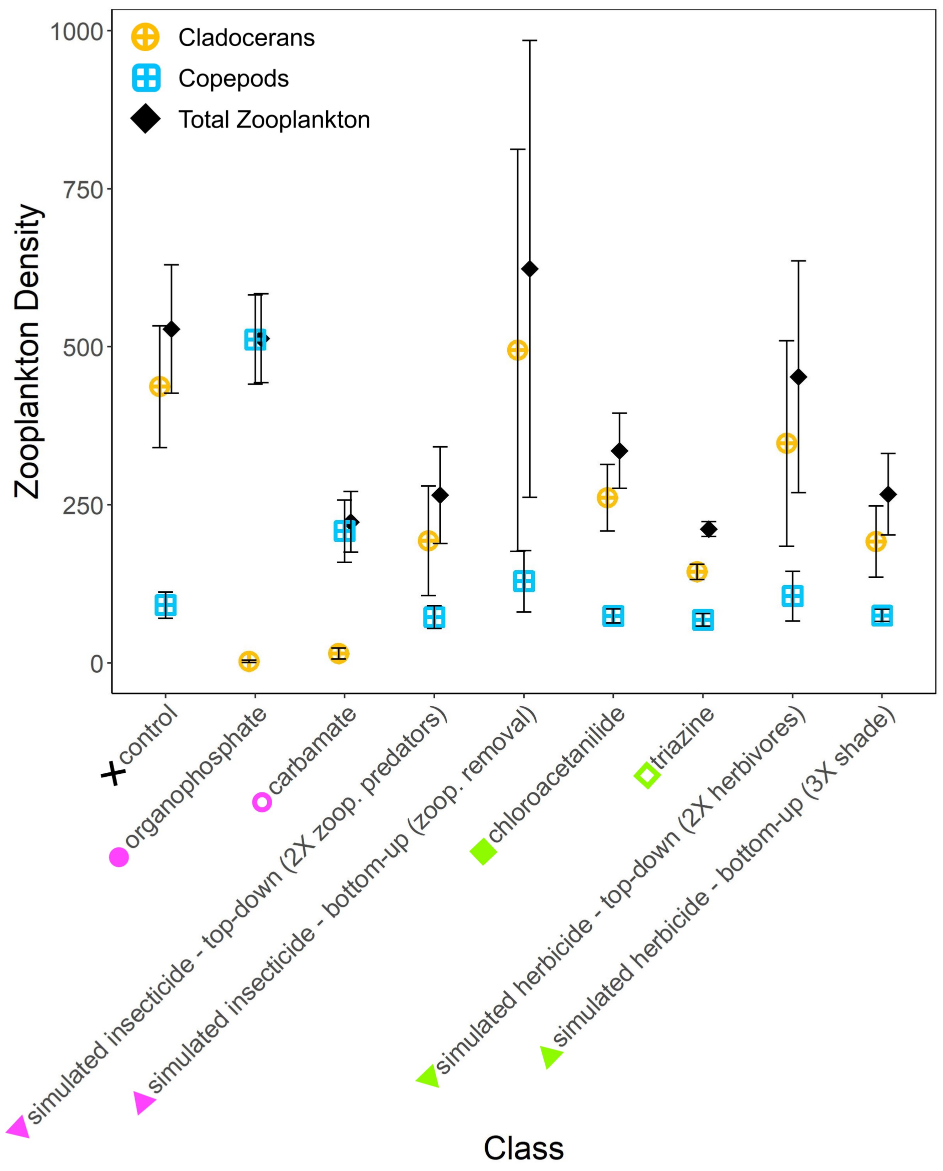

Supplementary Figure 3. Zooplankton densities in response to experimental treatments. Mean densities and standard errors of cladocerans, copepods, and total zooplankton in response to pesticide classes, simulated insecticides and herbicides, and the controls. Insecticide exposures lead to changes in zooplankton community composition with copepods becoming more abundant compared to cladocerans. In contrast, the main effect of herbicides on zooplankton was a decline in total abundance with no change in community composition; the relative amounts of cladocerans to copepods were comparable to the controls. Within a single experiment, the number of mesocosms within each treatments were as follows: n = 8 controls, n = 12 organophosphates, n = 12 carbamates, n = 4 top-down simulated insecticides, n =4 bottom-up simulated insecticides, n = 12 chloroacetanilides, n = 12 triazines, n = 4 top-down simulated herbicides, n = 4 bottom-up simulated herbicides.

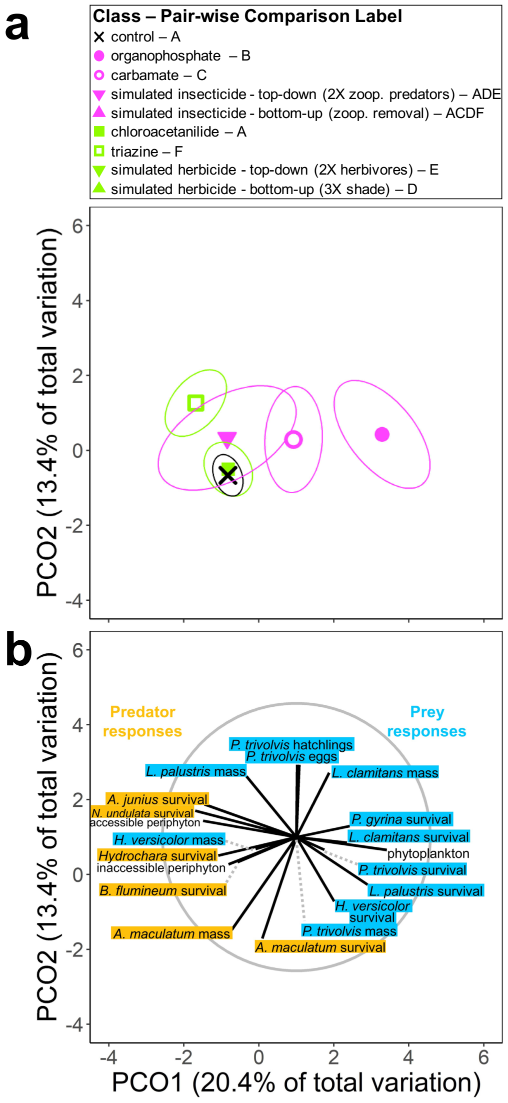

Supplementary Figure 4. Principal coordinates analysis for multivariate community responses. a Principal coordinates analysis plot of multivariate community-level responses, showing differences in pesticide treatments by type, class, and individual pesticide. Individual points are centroids for the representative treatment with ellipses based on 95% confidence intervals calculated using a standard error. Simulated herbicides and insecticides that were different from corresponding pesticide treatments, including bottom-up simulated insecticide and top-down and bottom-up simulated herbicides, were not included in the plot for ease of viewing. Pair-wise comparison labels are given in the figure legend. Treatments sharing letters are not different from each other. **b** Vector overlay of log-transformed and normalized community responses for corresponding principal coordinates analysis plot. Gray circle shows relative vector distance lengths; the gray circle corresponds to vector lengths that would have a correlation coefficient of one.

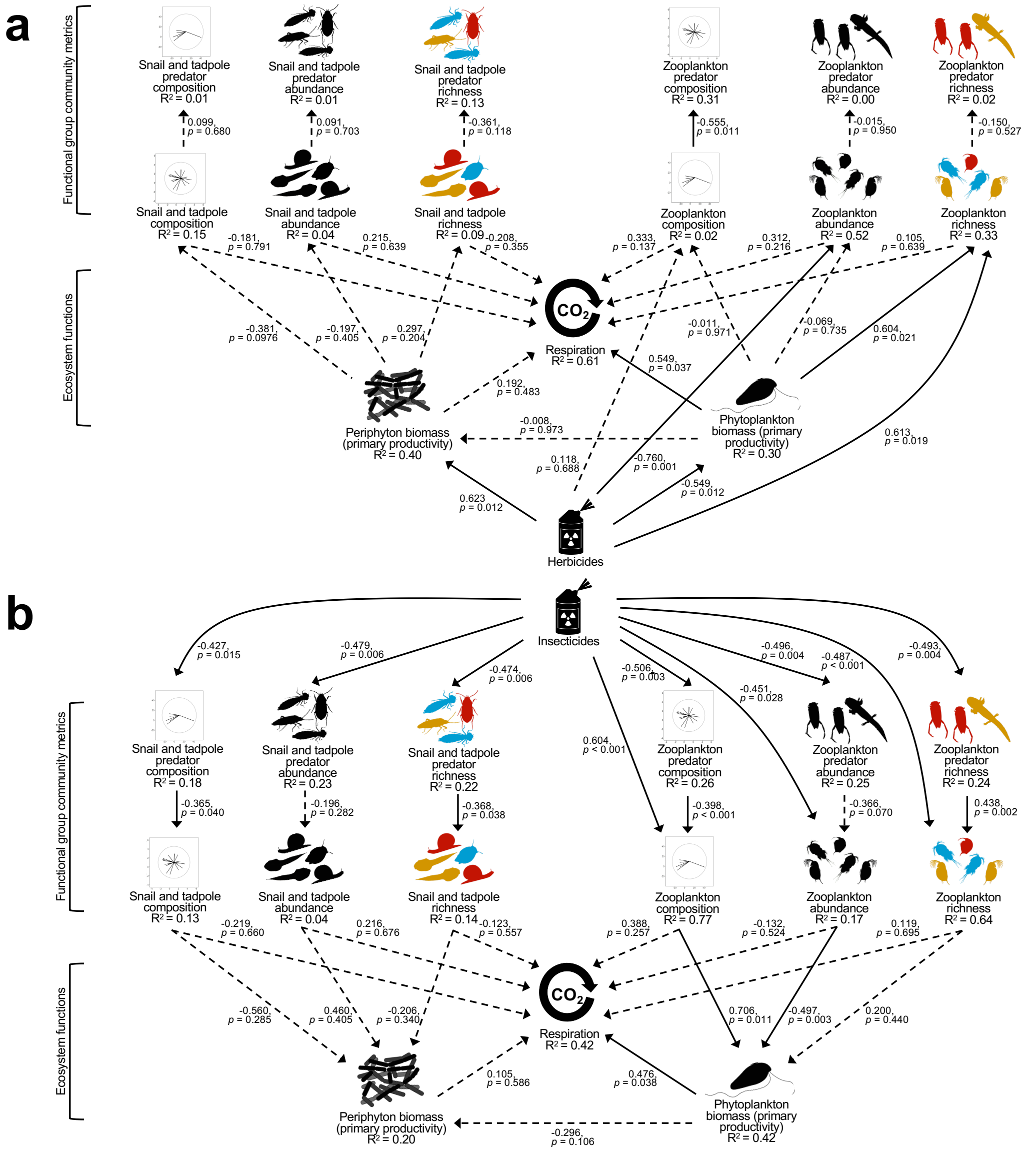

Supplementary Figure 5. Global structural equation models linking pesticide-induced changes in composition, abundance, and richness of functional groups to ecosystem functions. Non-significant paths of models were dropped, and final models are represented in the main text. **a** Through bottom-up effects, herbicide exposure decreases respiration through decreases in the abundance of phytoplankton, the most dominate functional group by biomass within the community. **b** The effects of insecticides on respiration and primary production of phytoplankton are driven by top-down effects on zooplankton composition and abundance, but not richness. Solid arrows are significant paths, and dotted arrows are non-significant paths. P-values, standardized coefficients, and conditional R^2 values are provided. In both **a** and **b,** the residuals of composition, abundance, and richness within a functional group covary. In addition for **b**, the residuals of the six predator variables covary. In both structural equation models, individual paths were linear models. Individual path *P*-values were based two-sided t-tests. The data fit the models well (**a**: Fisher's C = 3.601 with P-value = 0.165 on 2 degrees of freedom, **b**:

Fisher's C = 53.176 with P-value = 0.282 on 48 degrees of freedom). Attribution of silhouettes: periphyton (created by Matt Crook, license link, image has been rotated), anuran tadpole (created by M. Mahon (vectorization), J.J. Harrison (photography), photo license link, photo changed to silhouette), *A. junius* (created by M. Mahon (vectorization), Dave Huth (photography), photo license link, photo changed to silhouette), *B. flumineum* (created by Dave Angelini, license link, image was rotated and recolored), *Hydrochara* (created by T. Michael Keesey (vectorization), Yves Bousquet (photography), license link, image was rotated and recolored), and *P. gyrina* (created by M. Mahon (vectorization), N. Yotarou (photography), photo license link, photo changed to silhouette).

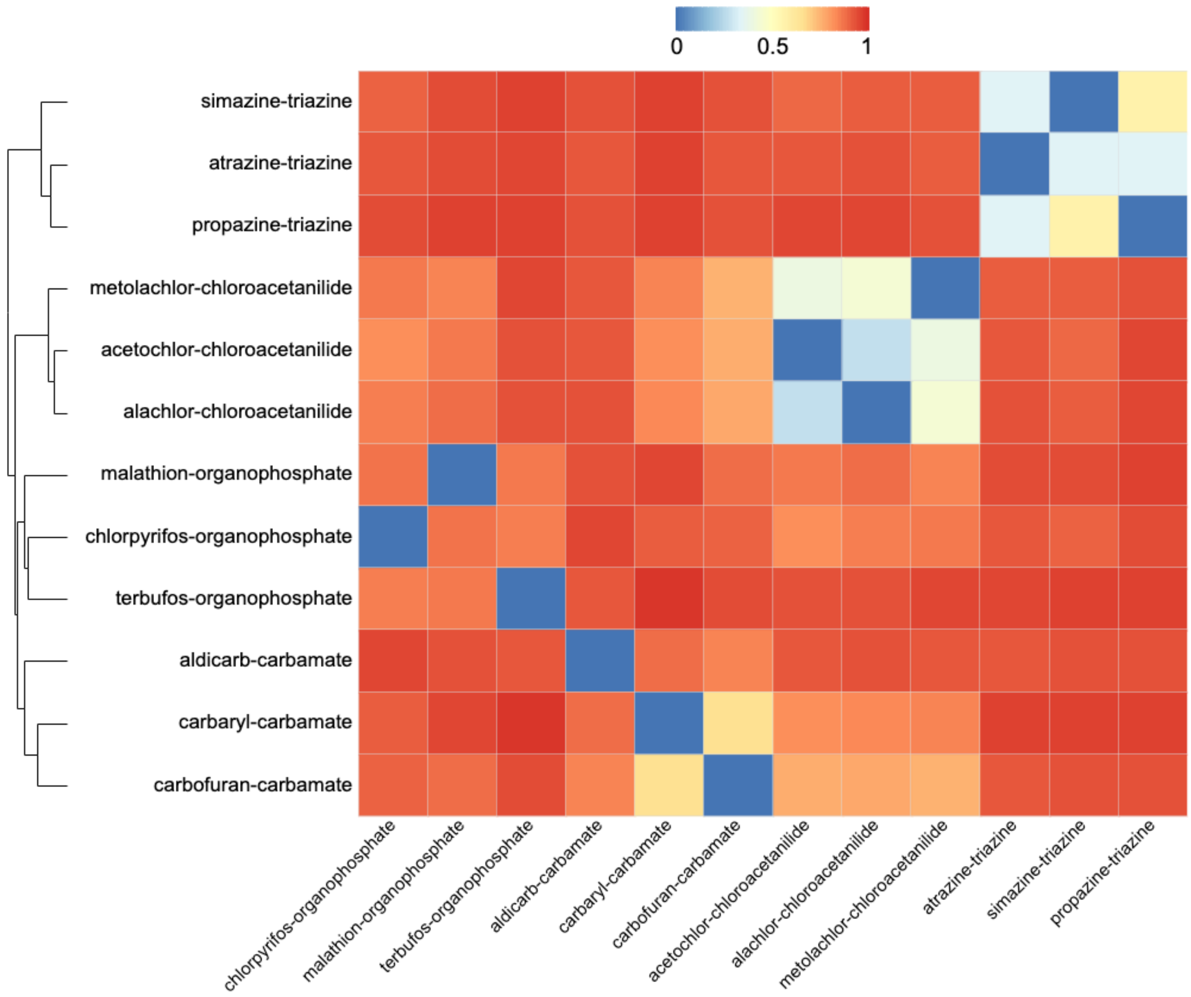

Supplementary Figure 6. Cluster diagram of pesticides by structural and physiochemical dissimilarities. Beside each pesticide is labeled with its class. The heat map is a measure of dissimilarity with 1 (red) indicating dissimilarity and 0 (blue) indicating similarity. Overall, pesticides in the same class are similar because they group together in the cluster diagram. Within classes, triazine herbicides and chloroacetanilide herbicides are more similar compared to organophosphate insecticides and carbamate insecticides within classes. Among classes, triazine herbicides are the most different compared to all other class, followed by organophosphate and carbamate insecticides, and chloroacetanilide herbicides are the most similar to other classes. While it is conceivable that chemical classes that are structurally similar may elicit similar effects on communities and ecosystems, our results do not support this prediction.

Supplementary Table 1. PERMANOVA model results. The effects of pesticides on multivariate responses, including ecosystem responses (pH, respiration, decomposition, turbidity, and biomasses of phytoplankton and accessible periphyton), zooplankton densities (densities of six genera), tri-trophic community responses (survival of all non-zooplankton species; average mass of surviving amphibians and *H. trivolvis* snails; average eggs and hatchlings of surviving *P. trivolvis* snails; periphyton; and phytoplankton), and simplified tri-trophic community (combined responses of algae, herbivores, and predators). All models account for the influence of a spatial block. *P* values were generated by Monte Carlo sampling and those less than 0.05 are bolded. Variation explained, represented as a proportion, is the estimated component of variation for a given predictor relative to the model's total variation, excluding block. So, variation explained accounts for the influence of the spatial block.

Endpoints and Source of Variation	*df*	Pseudo *F*	*p*	Variation Explained
Ecosystem				
Block	3	1.304	0.223	
Type	1	16.710	**0.004**	0.456
Class(Type)	2	2.075	0.068	0.117
Pesticide(Class(Type))	8	1.652	**0.037**	0.123
Residual	33			0.304
Zooplankton community				
Block	3	1.5395	0.124	
Type	1	9.6265	**0.020**	0.442
Class(Type)	2	4.551	**0.004**	0.188
Pesticide(Class(Type))	8	1.8831	**0.010**	0.118
Residual	33			0.252
Tri-trophic community				
Block	3	1.915	**0.005**	
Type	1	2.849	**0.038**	0.222
Class(Type)	2	1.806	**0.034**	0.154
Pesticide(Class(Type))	8	2.111	**0.0001**	0.215
Residual	33			0.409
Simplified tri-trophic community				
Block	3	2.697	**0.012**	
Type	1	4.271	0.087	0.291
Class(Type)	2	2.484	0.071	0.176
Pesticide(Class(Type))	8	1.924	**0.025**	0.173
Residual	33			0.360

Supplementary Table 2. Model parameters. Model parameters of GENEEC version 2 used to generate environmentally relevant pesticide concentrations (Peak EEC [estimated environmental concentration]) used in the experiment.

	Triazine herbicides			Chloroacetanilide herbicides			Carbamate insecticides			Organophosphate insecticides		
	Atrazine	Propazine	Simazine	Acetochlor	Alachor	Metolachlor	Aldicarb	Carbaryl	Carbofuran	Chlorpyrifos	Malathion	Terbufos
Model Parameter Inputs												
Trade name	Aatrex	Milocep	Princel 4L	Harness	Bullet	Dual II Magnum	Temik	Sevin 80S	Furadan	Dursban 50W	Fyfanon ULV	Counter 15G
Crop	Corn	Sorghum	Corn	Corn	Corn/ sorghum	Corn	Potatoes	Corn/ sorghum	Tobacco/ Barley	Turfgrass	Mosquito control	Corn
Application Rate (lbs a.i./acre)	2	2	4.4	3	2.8125	2.3875	3	2	1.624	8	6	7.395
Number of applications	1	1	1	1	1	1	1	4	1	1	1	1
Days between applications	-	-	-	-	-	-	-	7	-	-	-	-
K_d	-	-	1.96[a]	3.03[a]	-	-	0.053[a]	-	1.23[a]	-	-	-
K_{oc}	100[a]	65[d]	-	-	170[a]	200[a]	30[a]	300[a]	-	6070[a]	1248[b]	500[a]
Soil half-life (d)	300[b]	231[e]	100[c]	84[a]	49[c]	56[c]	72[a]	21[a]	120[a]	30.5[b]	6[c]	5[b]
Wetted application?	No	No	No	No	No	No	No	No	No	No	No	No
Application method	Ground spray	Ground spray	Ground spray	Ground spray	Ground spray	Ground spray	Granular (2 inches)	Ground spray	Aerial	Ground spray	Ground spray	Granular (surface)
No spray zone (ft)	0	0	0	0	0	0	0	0	0	0	0	0
Solutbility (mg/L)	33	8.5[b]	5[a]	223	242	530	6000[a]	40[a]	320[a]	2[a]	130[a]	5
Aquatic half-life (d)	742[c]	462[f]	700[c]	12[g]	98	-	10[a]	10[a]	57[a]	-	-	3.5[c]
Hydrolysis half-life (d)	-	-	-	-	-	210	-	-	-	78[c]	147[c]	-
Photolysis half-life (d)	335[d]	-	-	-	-	71	12[b]	45	5[b]	28[c]	-	-
Peak EEC (ppb)	102	106	202	123	127	105	91	219	209	64	101	171

a Exotoxnet
b USDA
c Spectrum Laboratories
d USEPA fact sheet
e Pesticide Action Network
f Two times the soil half-life
g http://pmep.cce.cornell.edu/profiles/herb-growthreg/24-d-butylate/acetochlor/new-ai-acetochlor.html

Supplementary Table 3. Attribution of images. Attribution, permission of use, and licenses for images in the main text and supplementary figures.

Image	Attribution	Permission of Use	License Type	Link to License	Notes
Periphyton	created by Matt Crook	link	CC BY-SA 3.0	link	Changes: image was rotated
Phytoplankton	created by T. Michael Keesey	link	CC0 1.0	link	
Helisoma (*Planorbella*) *trivolvis*	created by Scott Hartman	link	CC0 1.0	link	
Physa gyrina	created by Michael Mahon* (vectorization), N. Yotarou (photography)	link (photo)	photograph: CC BY-SA 3.0	link	Changes: photograph changed to silhouette
Anuran tadpole	created by Michael Mahon* (vectorization), J.J. Harrison (photography)	link (photo)	photograph: CC BY-SA 3.0	link	Changes: photograph changed to silhouette
Bosmina	created by Michael Mahon* (vectorization), S.F. Harmer and A.E. Shipley (photography)	link (photo)	photograph: Public Domain, copyright expired		link (book)
Diaphanosoma	created by Michael Mahon* (vectorization), Museo Civico di Storia Naturale di Genova (photography)	link (photo)	photograph: Public Domain, copyright expired		link (book)
Chydori	created by Michael Mahon* (vectorization), H.B. Ward and G.C. Whipple (photography)	link (photo)	photograph: Public Domain, copyright expired		link (book)
Daphnia	uncredited, submitted by Mike Keesey to PhyloPic Database	link	Public Domain Mark 1.0	link	
Cyclops	created by Michael Mahon* (vectorization), Great Lakes Image Collection (photography)	link (photo)	Public Domain, image taken by government employee (EPA)		

Diaptomus	created by Michael Mahon* (vectorization), NOAA Great Lakes Environmental Research Lab (photography),	link (photo)	Public Domain, image taken by government employee (NOAA)		
Anax junius	created by Michael Mahon* (vectorization), Dave Huth (photography)	link (photo)	photograph: CC BY 2.0	link	Changes: photograph changed to silhouette
Belostoma flumineum	created by Dave Angelini	link	CC BY 3.0	link	Changes: image was rotated
Hydrochara	created by T. Michael Keesey (vectorization), Yves Bousquet (photography)	link	CC BY 3.0	link	Changes: image was rotated
Ambystoma maculatum	created by Jake Warner	link	CC0 1.0	link	
Nototeca undulata	created by Michael Mahon* (vectorization); Christopher Johnson (photography)	link (photo)	CC0 1.0	link	
Pesticide spray can	created by Samantha Rumschlag* and Michael Mahon*				
Respiration symbol	created by Samantha Rumschlag*				
Composition symbol	created by Samantha Rumschlag*				

*author or co-author of the current manuscript

Supplementary Discussion

Performance of Simulated Pesticide Treatments

With the exception of bottom-up simulated herbicide, the effects of simulated pesticides did not match the effects of pesticide classes (pair-wise comparisons Fig. 1-3). These treatments performed poorly likely because manipulating taxa did not match the magnitude or the specificity of the long-term effect of pesticides. Top-down simulated herbicides (i.e., doubled herbivores) were designed to reduce algae, but the added tadpoles and snails mostly feed on periphyton, while the actual herbicides had a greater long-term net negative effect on phytoplankton (Fig. 1). Top-down (i.e., doubled zooplankton predators) and bottom-up (i.e., zooplankton removal) simulated insecticides both failed to replicate the differential toxicity that insecticides had on cladoceran versus copepod zooplankton (Supplementary Fig. 3).

Multigenerational Exposures

While multigenerational exposure of organisms to pesticides had the potential to alter vulnerability of organisms over time resulting in evolved tolerance or resistance to pesticide exposure, it seems unlikely that it played a large role in our study. Pesticide exposures might have only spanned multiple generations in treatments that included long-lived pesticides and for species with short generation times that could reproduce before the end of the four-week experiment. While we did not measure pesticide concentrations through time, we know from other studies of environmental persistence that soil half-lives of our chemical classes are ordered as follows: triazine herbicides (100-300 d), chloroacetanilides herbicides (49-84 d), carbamate insecticides (21-120 d), organophosphate insecticides (5-30.5 d) (see Supplementary Table 2). For the generation time of the community members, cladoceran zooplankton mature in three to six weeks (*4*) . Copepod zooplankton generally have even longer generation times compared to cladocerans, anywhere from 20-30 days in the tropics and up to one year in artic regions (*4*). Generation times of phytoplankton can be measured on the order of days (*5*). All other community members could not complete their life cycles in our mesocosms. Given the environmental persistence of the pesticides and the generation times of the organisms, it is likely that only phytoplankton could have possibly been exposed to pesticides across multiple generations. But, measurements of phytoplankton occurred only once during the experiment, so we do not suspect that changes in vulnerability of the organisms played a role in the observed patterns.

Structural Similarities Among Pesticide Classes

There is likely variation in the structural similarities among pesticide classes with some being more similar than others. Chemical classes that are structurally similar may elicit similar effects on ecosystems while chemical classes that are different may be more likely to have different effects. To determine the structural similarities of the 12 pesticides we used ChemMine Tools (https://chemminetools.ucr.edu/), which is an online service for analyzing how similar small molecules are. We used hierarchical clustering to evaluate how similar the 12 pesticides are in terms of structure and physiochemical properties. Similarity measures, calculated between pairs of pesticides, were atom pair and maximum common substructure (MCS) similarities derived from the Tanimoto coefficient. These atom pair similarity scores were transformed to generate pair-wise distance values, which were used in the hierarchical clustering.

The hierarchical clustering and distance matrix of the 12 pesticides (Supplementary Fig. 6). Overall, the figure shows that pesticides in the same class are similar because they group

together in the cluster diagram on the left, as one would expect. In addition, this figure supports that we had a range of structural similarities within and among chemical classes. Within classes, triazine herbicides and chloroacetanilide herbicides were more similar compared to organophosphate insecticides and carbamate insecticides within classes. Among classes, triazine herbicides seem the most different compared to all other class, followed by organophosphate and carbamate insecticides, and chloroacetanilide herbicides seem to be the most similar to other classes. So, one might predict that chloroacetanilides should most often be indistinguishable in their effects compared to the organophosphate and carbamate insecticides on aquatic communities and ecosystem responses. Our results do not align with this prediction. In the pairwise comparisons of the current manuscript, chloroacetanilides were only indistinguishable from triazines in the analyses of the zooplankton community (see Figure 2), even though the two classes are not very similar in structure). Our results support that type is more important than structure in explaining similarities or differences in effects. We also acknowledge that a different range of similarities across different pesticide classes could yield different results.

Evaluation of Acute Aquatic Toxicity Using QSAR Approaches

We completed analyses to evaluate if the consistency of pesticides on aquatic systems observed in the current study could be predicted by QSAR methods based on the structure of the pesticides alone. We used the QSAR Toolbox (https://qsartoolbox.org/) developed in partnership with The Organisation for Economic Co-operation and Development (OECD). QSAR Toolbox is a centralized, open-source software system for predicting toxicity of chemicals by applying a category approach. One functionality of the software is the clustering of chemicals into similar groups based on the predicted acute aquatic toxicity. The predicted acute aquatic toxicity is based on the pesticide's chemical structure. We conducted three different analyses that examined the clustering of our 12 pesticides. Below, we describe the three different analyses and the results.

1) Acute Aquatic Toxicity Classification by Verhaar

Description: The Acute aquatic toxicity classification by Verhaar consists of parametric and structural rules to mimic the Verhaar rules developed by Toxtree software. This system is introduced for chemical categorization purposes or can be used for the prioritization of chemicals for subsequent testing.

Results:
Group 1: All carbamates and organophosphates
Group 2: All triazines
Group 3: All chloroacetanilides

2) Acute Aquatic Toxicity by Mode of Action by OASIS

Description: This profile divides chemicals in different categories according to their acute toxic mode of action (MOA). 2D structural information is used only to identify the MOA of chemicals. Based on theoretical and empiric knowledge the following seven hierarchically ordered MOA are distinguished: Aldehydes; alpha, beta-Unsaturated alcohols; Phenols and Anilines; Esters; Narcotic Amines; Basesurface narcotics.

Results:

Group 1: All carbamates, organophosphates, and chloroacetanilides
Group 2: All triazines

3) Aquatic Toxicity Classification by ECOSAR
Description: The Aquatic Toxicity Classification by ECOSAR profiler consists of molecular definitions to mimic the structural definitions of chemical classes within the U.S. Environmental Protection Agency's Ecological Structure-Activity Program (ECOSAR™). ECOSAR™ contains a library of class-based structural activity relationships for predicting aquatic toxicity, overlaid with an expert decision tree based on expert rules for selecting the appropriate chemical class for evaluation of the compound.

Results:
Group 1: All triazines
Group 2: All chloroacetanilides
Group 3: Carbofuran and carbaryl (both carbamates)
Group 4: Aldicarb (carbamate)
Group 5: Terbufos (organophosphate)
Group 6: Malathion (organophosphate)
Group 7: Chlorpyrifos (organophosphate)

So, the result is that the pesticide groups vary based on the underlying assumptions of the model even though all three models are generally trying to predict pesticides that have similarity in the aquatic toxicities. These groupings are not very consistent with the observed toxicities to taxa in our study (tri-trophic and zooplankton communities). For instance, in the tri-trophic community analyses, the pairwise comparisons would suggest that all four pesticide classes behave differently. In the analyses of the zooplankton, the pairwise comparisons suggest that chloroacetanilides and triazines should group together while organophosphates and carbamates should form two additional separate groups.

The main issues with these groups of pesticides based on predicted toxicity is that they do not consider the variation across responses of groups of taxa and they do not consider indirect effects of pesticides. For instance, a QSAR model might predict direct effects of herbicides on algae, but that model will not include the indirect effect of herbicides on total zooplankton abundance.

Supplementary References

1. J. Travis, H. M. Wilbur, in *Ecological Eommunities: Conceptual Issues and Evidence* (Princeton, New Jersey, USA, 1984), pp. 113–122.

2. S. L. Rumschlag, N. T. Halstead, J. T. Hoverman, T. R. Raffel, H. J. Carrick, P. J. Hudson, J. R. Rohr, Effects of pesticides on exposure and susceptibility to parasites can be generalised to pesticide class and type in aquatic communities. *Ecol. Lett.* **22**, 962–972 (2019).

3. J. R. Rohr, A. M. Schotthoefer, T. R. Raffel, H. J. Carrick, N. Halstead, J. T. Hoverman, C. M. Johnson, L. B. Johnson, C. Lieske, M. D. Piwoni, P. K. Schoff, V. R. Beasley, Agrochemicals increase trematode infections in a declining amphibian species. *Nature*. **455**, 1235–1239 (2008).

4. J. Kalff, *Limnology: Inland Water Ecosystems* (Prentice Hall, 2002).

5. E. A. Laws, Evaluation of In Situ Phytoplankton Growth Rates: A Synthesis of Data from Varied Approaches. *Annu. Rev. Mar. Sci.* **5**, 247–268 (2013).
