# Supplementary material for: Consistent effects of pesticides on community structure and ecosystem function in freshwater systems
Source: Nat Commun. 2020 Dec 10;11:6333. doi: 10.1038/s41467-020-20192-2 (PMC7730384; doi:10.1038/s41467-020-20192-2)
Supplement: Supplementary file 2 — Reporting Summary [file 41467_2020_20192_MOESM2_ESM.pdf]

nature research

Corresponding author(s): Samantha Rumschlag

Last updated by author(s): Nov 13, 2020

Reporting Summary

Nature Research wishes to improve the reproducibility of the work that we publish. This form provides structure for consistency and transparency in reporting. For further information on Nature Research policies, see our Editorial Policies and the Editorial Policy Checklist.

Statistics

For all statistical analyses, confirm that the following items are present in the figure legend, table legend, main text, or Methods section.

n/a	Confirmed	
☐	☒	The exact sample size (*n*) for each experimental group/condition, given as a discrete number and unit of measurement
☐	☒	A statement on whether measurements were taken from distinct samples or whether the same sample was measured repeatedly
☐	☒	The statistical test(s) used AND whether they are one- or two-sided<br>*Only common tests should be described solely by name; describe more complex techniques in the Methods section.*
☐	☒	A description of all covariates tested
☐	☒	A description of any assumptions or corrections, such as tests of normality and adjustment for multiple comparisons
☐	☒	A full description of the statistical parameters including central tendency (e.g. means) or other basic estimates (e.g. regression coefficient) AND variation (e.g. standard deviation) or associated estimates of uncertainty (e.g. confidence intervals)
☐	☒	For null hypothesis testing, the test statistic (e.g. *F*, *t*, *r*) with confidence intervals, effect sizes, degrees of freedom and *P* value noted<br>*Give P values as exact values whenever suitable.*
☒	☐	For Bayesian analysis, information on the choice of priors and Markov chain Monte Carlo settings
☐	☒	For hierarchical and complex designs, identification of the appropriate level for tests and full reporting of outcomes
☒	☐	Estimates of effect sizes (e.g. Cohen's *d*, Pearson's *r*), indicating how they were calculated

Our web collection on statistics for biologists contains articles on many of the points above.

Software and code

Policy information about availability of computer code

Data collection	No data were collected with code in this study.
Data analysis	Pesticide concentrations were based on predictions from GENEEC v2. All PERMANOVA models, pair-wise comparisons, dbRDAs, PCoAs, and two-way cluster diagrams were executed using PERMANOVA+ for PRIMER version 7 (PRIMER-E Ltd, Plymouth, UK). For ease of visualization of dbRDA and PCoA plots, data from PERMANOVA+ for PRIMER were exported, and plots were made using 'ggplot2' package v3.2.1 in R. SEMs were constructed in R using piecewiseSEM v2.1.0. All analyses in R were conducted in v3.6.1. In the supplement, ellipses on the PCoA point plots were made using the 'vegan' package v2.5-6. QSAR analyses were completed using QSAR Toolbox v4.3

For manuscripts utilizing custom algorithms or software that are central to the research but not yet described in published literature, software must be made available to editors and reviewers. We strongly encourage code deposition in a community repository (e.g. GitHub). See the Nature Research guidelines for submitting code & software for further information.

Data

Policy information about availability of data

All manuscripts must include a data availability statement. This statement should provide the following information, where applicable:

- Accession codes, unique identifiers, or web links for publicly available datasets
- A list of figures that have associated raw data
- A description of any restrictions on data availability

Data used in the generation of pesticide concentrations used in the present experiment are available within supplementary information. All other relevant data supporting the findings of this study are available at Figshare (doi.org/10.6084/m9.figshare.13224587).

Field-specific reporting

Please select the one below that is the best fit for your research. If you are not sure, read the appropriate sections before making your selection.

☐ Life sciences ☐ Behavioural & social sciences ☒ Ecological, evolutionary & environmental sciences

For a reference copy of the document with all sections, see nature.com/documents/nr-reporting-summary-flat.pdf

Ecological, evolutionary & environmental sciences study design

All studies must disclose on these points even when the disclosure is negative.

Study description	We conducted a randomized-block experiment with replicated mesocosm ponds. We randomly assigned 18 treatments (12 pesticides, 4 simulated pesticides, 2 controls) with four replicate mesocosms of each treatment, which resulted in 72 total mesocosms (Fig. 1a). The 12 pesticide treatments were nested; we included two pesticide types (insecticide, herbicide), two classes within each pesticide type (organophosphate insecticide, carbamate insecticide, chloroacetanilide herbicide, triazine herbicide), and three different pesticides in each of four classes.
Research sample	The experimental replicate was a mesocosm pond.
Sampling strategy	Each pesticide treatment was replicated four times. This sample size was the largest our experimental team could logistically handle given the experimental design and question. This determination of sample size was based on previous personal experience. No statistical analyses were completed to determine sample size. We measured decomposition by taking the dry mass of hardwood leaf packets in each mesocosm at the beginning and the end of experiment. We measured pH and dissolved oxygen (DO) at dusk and dawn on subsequent days using hand-held meters (YSI, Yellow Springs, OH, USA). Turbidity was based on a visual clarity score. We sampled periphyton using clay tiles (100 cm2) oriented perpendicularly along the bottom of the mesocosm. We sampled phytoplankton from water samples taken 10 cm below the water surface. Zooplankton were collected from the entire water column by placing a PVC pipe (10 cm diameter, 60 cm height) upright in the center of each tank, capping the bottom, and pouring the water through a 20 μm Nitex mesh. We sampled snail egg masses and hatchlings using two rectangular pieces of Plexiglass (465 cm2) in each mesocosm, one hung on the side and one on the bottom of the mesocosm. At the end of the experiment, we calculated survival of tadpoles, salamanders, snails, and insects. We weighed surviving tadpoles. Additional details on sampling methods are contained within the manuscript.
Data collection	Data were collected in teams that rotated the identity of who was collecting and recording the data. Data were collected in field notebooks.
Timing and spatial scale	The experiment ran for four weeks, from 18 June to 13 July 2007. The spatial scale was a single outdoor mesocosm field at the Russell E. Larsen Agricultural Research Center (Pennsylvania Furnace, PA, USA). Each response was measured once throughout the experiment. Turbidity, phytoplankton, periphyton, and zooplankton were measured on 2 July 2007. DO and pH were measured on 10 July 2007. Survival and mass of organisms, snail eggs and hatchlings, and final mass of hardwood leaf packets was measured on 13 July 2007. The timing of sampling was based on visual changes in the algal communities and logistical constraints of the research team.
Data exclusions	No data were completely excluded from all analyses. As noted in the paper, mesocosms treated with chloroacetanilide herbicides were excluded from the analysis because they did not influence algal densities. This exclusion of chloroacetanilides from the SEMs was not pre-established. It is important to note that chloroacetanilides were included in all other statistical analyses.
Reproducibility	Based on the size and length of time of the experiment, it was not possible to repeat the experiment.
Randomization	Mesocosms were randomized by treatment across the experimental field.
Blinding	All researchers were blinded to treatment for data collection during the study.

Did the study involve field work? ☐ Yes ☒ No

Reporting for specific materials, systems and methods

We require information from authors about some types of materials, experimental systems and methods used in many studies. Here, indicate whether each material, system or method listed is relevant to your study. If you are not sure if a list item applies to your research, read the appropriate section before selecting a response.

Materials & experimental systems

n/a	Involved in the study	
☒	☐	Antibodies
☒	☐	Eukaryotic cell lines
☒	☐	Palaeontology and archaeology
☐	☒	Animals and other organisms
☒	☐	Human research participants
☒	☐	Clinical data
☒	☐	Dual use research of concern

Methods

n/a	Involved in the study	
☒	☐	ChIP-seq
☒	☐	Flow cytometry
☒	☐	MRI-based neuroimaging

Animals and other organisms

Policy information about studies involving animals; ARRIVE guidelines recommended for reporting animal research

Laboratory animals	No laboratory animals were used in this experiment.
Wild animals	The only vertebrates used in the experiment included 3 Ambystoma maculatum, 20 Hyla versicolor, 20 Lithobates palustris, and 20 Lithobates clamitans per mesocosm. All vertebrates were collected as aquatic larvae. It is impossible to sex amphibians at this stage of development without dissection. Invertebrates used in the experiment included 11 Helisoma (Planorbella) trivolvis adults, 10 Physa gyrina adults, 2 Anax junius larvae, 2 Belostoma flumineum adults, 5 Hydrochara sp. adults, and 6 Nototeca undulata adults per mesocosm. We did not record the sex of these invertebrates. All animals were collected in local ponds with a net and were transported and held in pond water and fed ad libitum until the start of the experiment. At the end of the study, amphibians were euthanized using MS-222 , and invertebrates were euthanized using ethanol. Animals were euthanized so they could be stored and preserved long-term.
Field-collected samples	No field samples were used in the study.
Ethics oversight	Ethics oversight was provided by the Penn State University IACUC.

Note that full information on the approval of the study protocol must also be provided in the manuscript.
